# Regulation of Garcinol on Histone Acetylation in the Amygdala and on the Reconsolidation of a Cocaine-Associated Memory

**DOI:** 10.3389/fnbeh.2019.00281

**Published:** 2020-01-08

**Authors:** Melissa S. Monsey, Sonia G. Ruiz, Jane R. Taylor

**Affiliations:** ^1^Department of Psychiatry, Yale School of Medicine, New Haven, CT, United States; ^2^Department of Psychology, Yale University, New Haven, CT, United States; ^3^Department of Neuroscience, Yale University, New Haven, CT, United States

**Keywords:** garcinol, reconsolidation, histone acetylation, amygdala, cocaine-cue memory

## Abstract

Exposure to drug-related cues often disrupts abstinence from cocaine use by triggering memories of drug effects, leading to craving and possible relapse. One prospective method of treatment is weakening cocaine-associated memories *via* impairment of memory reconsolidation. Previous experiments have shown that systemic injection of the amnestic agent garcinol impairs the reconsolidation of cocaine-cue memories in a temporally constrained, cue-specific, and persistent manner. Here, we investigated garcinol’s effect on cocaine-cue memory reconsolidation when administered to the lateral nucleus of the amygdala (LA), as well as its epigenetic activity following systemic garcinol administration and also when given in conjunction with trichostatin A (TSA), a histone deacetylase (HDAC) inhibitor. Rats received 12 days of cocaine self-administration training during which time an active lever press resulted in an i.v. cocaine infusion that was concurrently paired with the presentation of a light/tone cue. After 8 days of lever extinction, rats received a memory reactivation session followed by a cue-induced reinstatement test. Intra-LA garcinol following memory reactivation significantly impaired reconsolidation only if the memory was reactivated. Additional studies revealed a significant reduction in histone H3 K27 acetylation and reduced expression of the immediate-early genes Arc and Egr-1 in the LA. When administered alone, TSA enhanced the reinstatement of a cocaine-cue memory, an effect that was prevented when garcinol was concurrently administered. These data indicate the LA is a key structure responsive to garcinol, suggest that one of garcinol’s mechanisms of action is through the reduction of memory-related gene expression in the LA, implicate changes in histone acetylation in memory reconsolidation, and support garcinol as a potential therapeutic tool for sustaining abstinence.

## Introduction

One commonality among substance use disorders is the tendency to relapse to drug-seeking behaviors that is a major detrimental factor for long-term abstinence. Much research has been focused on identifying novel neural mechanisms that underlie craving and drug-seeking behavior in an effort to develop more effective treatments. One important contributing factor that may initiate relapse is exposure to environments or cues that have become associated with drug-taking. These cues can elicit memories of the pleasurable effects of taking the drug and ultimately result in craving and drug-seeking behavior, preventing sustained abstinence. Therefore, one potentially therapeutic treatment option is to identify new mechanisms and methods to weaken the strength of these drug-associated memories.

Reconsolidation and extinction have been widely identified as two primary processes by which existing memories can be modified. Although extinction or exposure-based treatment methods are promising, used alone they have failed to be successful (Taylor et al., 2009; Torregrossa and Taylor, 2013; Everitt et al., 2018). Memory extinction is the process whereby repeated exposure to the conditioned stimulus (CS) is performed in a context lacking the unconditioned stimulus (US). Following multiple exposures to the CS, a new memory is formed in which the CS no longer elicits the conditioned response (Kindt et al., 2009). In a clinical setting, this consists of repeatedly exposing patients to the cues. Such processes can be anxiety-provoking for patients; furthermore, extinction learning may actually be impaired in patients with psychiatric disorders (Holt et al., 2009; Singewald and Holmes, 2019). In addition, memories that are successfully extinguished are not “erased”; they are susceptible to spontaneous recovery with the passage of time, are capable of being reinstated during periods of stress (Singewald et al., 2015; Mantsch et al., 2016), and may renew in new contexts other than that in which the memory was extinguished (Crombag and Shaham, 2002; Kindt et al., 2009).

During the process of reconsolidation, a memory that has previously been consolidated is recalled and subsequently enters a destabilized state for a short period of time, allowing the memory to be updated with new information before becoming restabilized, which can either strengthen or weaken the original memory (Zhang et al., 2018; Kida, 2019). Importantly, when memory is disrupted through interference with the reconsolidation process, the impairment appears to be persistent and is not prone to the constraints of extinction (Kindt et al., 2009; Singewald et al., 2015; Kida, 2019). In light of this, it has been suggested by researchers that “reconsolidation”-based therapy may be more clinically effective and less stressful. Utilizing behavioral strategies and/or pharmacological methods to interfere with this process has been shown to successfully impair the reconsolidation of cue memories in animals (Lewis, 1979; Nader, 2003; Lee et al., 2005, 2006, 2017; Tronson and Taylor, 2007; Sanchez et al., 2010; Sorg, 2012; Xue et al., 2012; Sartor and Aston-Jones, 2014; Taylor and Torregrossa, 2015; Torregrossa and Taylor, 2016; Dunbar and Taylor, 2017a; Monsey et al., 2017; Haubrich and Nader, 2018).

It is widely accepted that associative memories, such as a cocaine-cue memory, are formed and stored in an important brain region, the lateral nucleus of the amygdala (LA), which has therefore been the target of much research (Thomas et al., 2003; Lee et al., 2005, 2006; Tipps et al., 2014; Rich et al., 2016, 2019). Numerous studies have demonstrated that interfering with signaling cascades within the LA impairs its ability to form and store cocaine-associated memories (Wan et al., 2014; Shi et al., 2015; Rich et al., 2016, 2019).

Previous work from our lab has indicated that one such compound, the amnestic agent garcinol, can impair the reconsolidation of a cocaine-cue memory in a manner that requires memory reactivation, temporally regulated, long-lasting, persistent after extended access to cocaine, and cue-specific (Monsey et al., 2017). Further studies showed garcinol can also impair conditioned reinforcement learning, weakens the ability of acquiring a new response, and can also impair reinstatement following a US (cocaine) reactivation session (for review see Dunbar and Taylor, 2017a,b; Monsey et al., 2017).

Garcinol, a natural compound derived from the fruit rind of *Garcinia indica*, the Kokum tree, has been investigated in therapeutic contexts as a treatment for AIDS, HIV, and cancer (Yamaguchi et al., 2000; Koeberle et al., 2009; Padhye et al., 2009; Ahmad et al., 2010). Studies have found garcinol to be a potent histone acetyltransferase (HAT) inhibitor of the transcriptional coactivator p300 (EP300 binding protein)/CBP (CREB-binding protein) family, and PCAF (p300/CBP-associated factor) family; p300/CBP activity has been found to play a key role in the reconsolidation of auditory fear memories (Maddox et al., 2013a; Merschbaecher et al., 2016).

Garcinol is also of interest because of its role in modulating epigenetic processes in the LA, for example, histone acetylation (Ac), which may underlie memory reconsolidation mechanisms (Maddox and Schafe, 2011; Monsey et al., 2011; Maddox et al., 2013a,b; Hitchcock et al., 2019). Memory reactivation also has been shown to regulate levels of histone H3 protein in the amygdala (Maddox and Schafe, 2011). Further, administration of a histone deacetylase (HDAC) inhibition in the LA following memory reactivation enhances reconsolidation of an auditory fear memory, while use of a HAT inhibitor, like the amnestic agent garcinol, impairs memory reconsolidation (Maddox et al., 2013a,b).

These epigenetic mechanisms are thought to play an important role in the reconsolidation of auditory fear memories, yet little is known about their involvement in the reconsolidation of appetitive memories and drug-associated memories in particular. Thus, we explore the effect of the HAT inhibitor garcinol, on reconsolidation of cocaine-associated cue memories.

## Materials and Methods

### Subjects

Adult male Sprague–Dawley rats (Charles River), aged 2–3 months and weighing 275–300 g, were singly housed and kept on a 12 h light/dark cycle. Following recovery from surgery, food was restricted for the duration of the experiment to maintain rats at 90–95% of their pre-surgery body weight. Water was provided *ad libitum*.

### Surgical Procedures

Rats were anesthetized with a mixture of ketamine (75 mg/kg) and Xylazine (5 mg/kg, i.p.). They also received 5 mg/kg Rimadyl and 5 ml s.c. of lactated Ringer’s solution. Indwelling catheters were implanted into the right jugular vein. Catheters were perfused with heparinized saline every other day to maintain patency. For intra-cranial infusion experiments, during the same surgery immediately following the catheterization, rats were implanted bilaterally with 26-gauge stainless steel guide cannulas that were aimed at the LA (Bregma −3.2 AP, ±5.0 ML, −8.0 DV). The cannulas were adhered to several screws in the skull using a mixture of dental cement and acrylic. Dummy cannulas (31-gauge) were inserted into the guide cannulas to keep them from clogging. Following surgery, rats received 1 week of recovery time where they were singly housed with provided with *ad libitum* food and water. Rats were weighed daily throughout the remainder of all experiments.

### Behavioral Procedures

For self-administration training, rats were placed in sound-attenuated operant conditioning chambers (Med Associates). The boxes contained two extendable levers (on the same wall), a cue light, a separate house light, a speaker for the tone, and a background noise-generating fan.

Rats received 12 days of cocaine self-administration (SA) training occurring in 1-h sessions. Throughout the session, an active and inactive lever was extended. Each active lever press resulted in immediate i.v. infusion of cocaine (1 mg/kg) while simultaneously a cue light and tone (75 dB) were presented in the chamber for 10 s. An inactive lever press did not result in cocaine infusion or cue presentation. For self-administration training a fixed ratio 1 (FR1) schedule was used; one active lever press = 1 cocaine infusion/cue presentation. Rats then underwent 8 days of lever extinction, where pressing either lever had no outcome.

Rats were required to meet acquisition criteria of ≥6 infusions for each of the last 3 days of self-administration. This criteria, on average, is met by 90–95% or rats. These rats were then divided into to-be-vehicle or to-be-garcinol groups and balanced for a total number of infusions over all the days of SA and comparable levels of extinction. Twenty-four hours after the last extinction day, rats were placed in a novel chamber (addition of a novel lemon-scented odor, changes in floor texture, and different lighting) for a memory reactivation session. Here, rats received three presentations of the light and tone cues recall the cocaine-cue memory. There were no levers present. For no-reactivation controls, rats were placed in the same novel chamber, however, they did not receive cue presentation. For studies using systemic administration of garcinol or vehicle, rats received a 10 mg/kg i.p. injection 30 min after reactivation (and an additional injection of 2.5 mg/kg trichostatin A (TSA) or vehicle 45 min after reactivation in rescue experiment) and were returned to the animal colony. In experiments using intra-LA infusion of garcinol or vehicle, rats received a 500 ng 0.5 μl/side infusion 1 h after reactivation and were returned to the animal colony. For qRT-PCR experiments, rats were sacrificed 1 h after reactivation (30 min after garcinol or vehicle treatment) and brains were stored at −80°C until processed. In behavioral studies, rats were tested for cue-induced reinstatement 24 h after reactivation in the original chamber. During this test, an active lever press resulted in a 10 s light/tone cue presentation but did not result in cocaine infusion.

### Quantitative Real-Time PCR

Punches were taken on a sliding freezing microtome from the LA using a 1 mm punch tool from 400 μm thick sections. For RNA isolation, samples were processed using an RNAqueous Micro Kit (ThermoFisher). For cDNA synthesis, a High Capacity cDNA Reverse Transcription Kit (Applied Biosystems) was used. Quantitative real-time PCR (qRT-PCR) was performed using the ΔΔCt method using custom primers (Integrated DNA Technologies, Coralville, IA, USA) for Arc (Forward CCCTGCAGCCCAAGTTCAAG; Reverse GAAGGCTCAGCTGCCTGCTC) and Egr-1 (Forward AGCGAACAACCCTATGAGCA; Reverse TCGTTTGGCTGGGATAACTC). Relative gene concentrations were normalized to GAPDH (Forward GCATCCTGCACCACCAACTG; Reverse ACGCCACAGCTTTCCAGAGG). Data were analyzed using a two-tailed *t*-test with a significance threshold of *p* < 0.05. Data are normalized to GAPDH and then expressed as the average threshold cycle (Ct) difference between groups. Average fold change values were then calculated and values were expressed as a percentage of the control.

### Western Blotting

For Western blotting, 400-μm-thick sections of LA were cut on a sliding freezing microtome and 1 mm punches were taken. Tissue was manually dounced in 150 μl of ice-cold lysis buffer [4.39 g sucrose, 1 M HEPES, 0.5 M EDTA, 1 M NaF, 350 mM NaVO, and 1 protease inhibitor cocktail tablet (Sigma)]. Homogenates were electrophoresed on 4–20% gels (Criterion TGX) and blotted to 0.2 μm Midi PVDF membrane (Bio-Rad, Hercules, CA, USA). Western blots were blocked in 5% bovine serum albumin (BSA; Sigma Fraction V, Catalog #A-9647) in 1× Phosphate-Buffered Saline with Tween-20 [PBST; 10× Phosphate-Buffered Saline Solution Concentrate (1.4 M NaCl, 0.1 M phosphate pH 7.4, 0.03 M KCl), Milli-Q water, and 0.05% Tween-20 pH 7.4] then incubated with anti-histone H3-acetyl 18 (1:1,000; Abcam, ab1191) and anti-histone H3-acetyl 27 (1:500; Abcam, ab4729) antibody. Blots were then incubated with anti-rabbit conjugated to horseradish peroxidase (1:10,000; Vector Laboratories, Inc., Burlingame, CA, USA) and developed using Western Lightning Plus-ECL Enhanced Chemiluminescence Substrate (PerkinElmer). GAPDH (1:2,500; Abcam, ab9485) was used as a loading control for these experiments. Optical densities of bands were analyzed using ChemiDoc Molecular Image software (Bio-Rad). For analysis of AcH3K18 and AcH3K27, densities were expressed as a percentage of the vehicle control group.

### Statistical Analysis

Self-administration data were analyzed by repeated measure (RM) analysis of variance (ANOVA) across each day for total infusions, total active and inactive lever presses. Extinction training was also analyzed using ANOVA across each day to measure total active and inactive lever responses. Reinstatement tests were analyzed using RM-ANOVAs measuring total active and inactive lever responses on the last day of extinction and reinstatement test day. Bonferroni adjustment and *post hoc* tests were used where appropriate. qRT-PCR and Western blotting data were analyzed with two-tailed *t*-tests.

## Results

### Intra-LA Garcinol Impairs Cue-Induced Reinstatement of a Cocaine-Associated Memory Following Memory Reactivation

**Figure 1:**
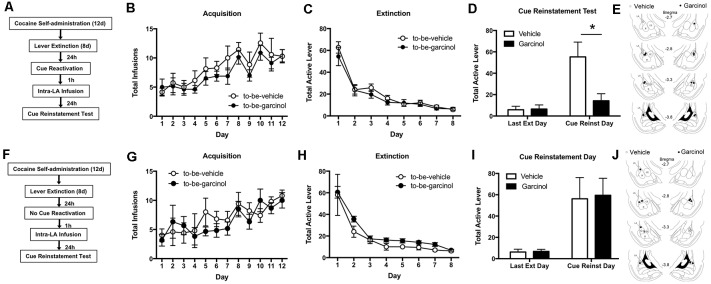
Intra-lateral nucleus of the amygdala (LA) garcinol impairs cue-induced reinstatement of a cocaine-associated memory following memory reactivation. **(A)** Schematic of the behavioral protocol for reactivated rats. **(B)** Total infusions per group across each day of cocaine self-administration. **(C)** Total active lever presses across each day of extinction. **(D)** Total active lever presses on the last day of extinction compared to during the cue-induced reinstatement test. *p < 0.05, significant decrease relative to vehicle group. **(E)** Verification of cannula placements for rats infused with vehicle (white circles) and garcinol (black circles). **(F)** Schematic of the behavioral protocol for non-reactivated rats. **(G)** Total infusions per group across each day of cocaine self-administration. **(H)** Total active lever presses across each day of extinction. **(I)** Total active lever presses on the last day of extinction compared to during the cue-induced reinstatement test. **(J)** Verification of cannula placements for rats infused with vehicle (white circles) and garcinol (black circles). *p < 0.05.

In our first experiment we examined whether the intra-LA infusion of garcinol impairs the reconsolidation of a cocaine-associated memory following either memory reactivation or in no reactivation controls (see Figure 1A). Here, rats received 12 days of cocaine self-administration training where each active lever press resulted in a 1 mg/kg i.v. infusion of cocaine as well as the presentation of a light/tone cue at the same time. Following this, rats underwent 8 days of lever extinction where active lever presses no longer resulted in cocaine infusion or cue presentation. Twenty-four hours after the last day of extinction, rats underwent a reactivation session in a novel context where the cue was presented. No differences were seen in the acquisition of the self-administration task between to-be-vehicle (*N* = 7) and to-be-garcinol (*N* = 8) groups. We report no significant differences in the number of total cocaine infusions between these groups (*p* > 0.05; Figure 1B). There were also no differences during lever extinction between groups for total active lever presses (*p* > 0.05; Figure 1C). The ANOVA across the last final extinction day and the cue reinstatement test day revealed a significant main effect of day (*F*_(1,13)_ = 102, *p* < 0.0001) and of drug (*F*_(1,13)_ = 46.79, *p* < 0.0001) as well as a significant interaction in active lever presses between garcinol (500 ng/side; 0.5 μl) and vehicle infused rats (*F*_(1,13)_ = 54.21, *p* < 0.0001; Figure 1D). We found that post-reactivation intra-LA garcinol decreased active lever pressing during the reinstatement test when compared to vehicle controls [*p* < 0.05 (see Figure 1E for estimates of infusion sites)]; however, no differences were observed between groups on the last day of extinction. Bonferroni’s test revealed a significant difference in vehicle infused rats from the last day of extinction compared to the reinstatement day (*p* < 0.0001), while no significant difference was observed from extinction to reinstatement day in garcinol injected rats (*p* > 0.05). This suggests that intra-LA, like systemically administered, garcinol can also block the reconsolidation of a cocaine-cue memory to decrease drug-seeking behavior.

To enhance its clinical utility, it is important to show that garcinol only blocks memories that have been reactivated and not those that have not. To control for this, we next examined whether garcinol would have an effect on a cocaine-cue memory receiving “no-reactivation” (see Figure 1F). Here, a second group of rats went through self-administration training and lever extinction as in our reactivation experiment. However, on the reactivation day, rats were placed in the reactivation chamber for the same length of time as the reactivated groups, but they were not exposed to cue presentations. One hour after this session rats received an injection of either vehicle or garcinol, as above, and then underwent a cue reinstatement test 24 h later. Similar to our reactivation experiments, we found no differences in the number of infusions between to-be-vehicle (*N* = 5) and to-be-garcinol (*N* = 5) groups during acquisition of cocaine self-administration (*p* > 0.05; Figure 1G). Likewise, there were no differences in active lever presses between groups throughout lever extinction (*p* > 0.05; Figure 1H). The ANOVA for the reinstatement test day compared to the last day of lever extinction revealed a significant main effect of day (*F*_(11,99)_ = 5.38, *p* < 0.01), but a nonsignificant main effect of group and no significant interaction between garcinol and vehicle-injected groups [*p* > 0.05; Figure 1I (see Figure 1J for estimates of infusion sites)]. These findings suggest that the LA is a key region involved in garcinol’s effects on cocaine-cue memory reconsolidation. Moreover, because garcinol does not alter reinstatement in the absence of memory reactivation, these results also suggest that garcinol’s effects on reconsolidation are predicated on active reactivation of cocaine-associated memory.

### Systemic Garcinol Decreases the Expression of Immediate-Early Genes in the LA of Reactivated Rats

**Figure 2:**
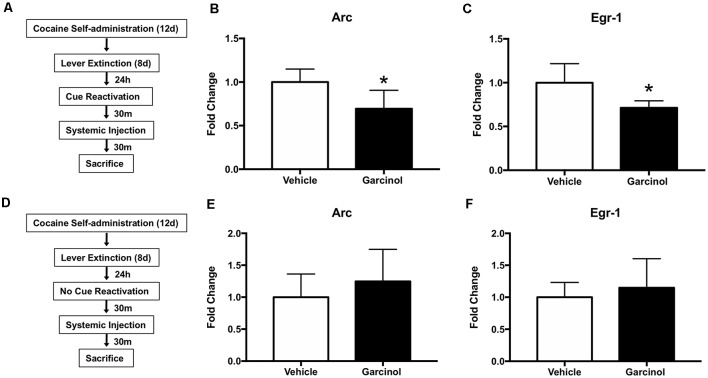
Systemic garcinol blocks the expression of immediate-early genes in the LA of reactivated rats. **(A)** Schematic of the behavioral protocol. **(B)** Quantification of Arc mRNA in the LA in vehicle and garcinol treated rats following memory reactivation using qRT-PCR. *p < 0.05, significant decrease relative to vehicle group. **(C)** Quantification of Egr-1 mRNA in the LA in vehicle and garcinol treated rats following memory reactivation using qRT-PCR. *p < 0.05, significant decrease relative to vehicle group. **(D)** Schematic of the behavioral protocol. **(E)** Quantification of Arc mRNA in the LA in vehicle and garcinol treated rats following no memory reactivation using qRT-PCR. **(F)** Quantification of Egr-1 mRNA in the LA in vehicle and garcinol treated rats following no memory reactivation using qRT-PCR. *p < 0.05.

Our next set of experiments examined the expression of genes previously shown to be regulated by memory reactivation in the LA and the effects of garcinol on expression of these genes (Figure 2; Maddox et al., 2010; Maddox and Schafe, 2011; Ziółkowska et al., 2011; Alaghband et al., 2014). Of the many genes involved in the consolidation and reconsolidation of long-term memories, several immediate-early genes (IEGs) are quickly transcribed in response to powerful external stimuli, such as fear conditioning. This first wave of gene expression is thought to be key for the later consolidation of the memory trace, ultimately leading to structural (i.e., morphological) changes at LA synapses. Here, we chose to examine the IEGs Arc and Egr-1, as they have been shown to be required for memory reconsolidation processes (Thomas et al., 2003; Lee et al., 2004, 2006; Maddox et al., 2010; Maddox and Schafe, 2011; Everitt, 2014) and are enhanced in the LA following reactivation of a drug-associated cue in a cocaine self-administration model (Ziółkowska et al., 2011).

For this set of experiments rats received systemic vehicle or garcinol (10 mg/kg, i.p.) administration following memory reactivation (vehicle *N* = 5; garcinol *N* = 6; see Figure 2A) or no reactivation (vehicle *N* = 5; garcinol *N* = 6; see Figure 2D). Again, there were no differences in cocaine infusions between to-be-vehicle or to-be-garcinol groups across self-administration (*p* > 0.05) or across extinction sessions in the reactivated and non-reactivated groups (*p* > 0.05; Supplementary Figure S1). Rats were sacrificed 30 min following reactivation or no reactivation and LA tissue was processed for qRT-PCR. The results revealed a significant reduction in Arc (*t*_(9)_ = 2.71, *p* < 0.05; Figure 2B) and Egr-1 (*t*_(9)_ = 3.03, *p* < 0.05; Figure 2C) mRNA in rats that received garcinol treatment following cocaine-cue memory reactivation. Conversely, in rats receiving no memory reactivation, we did not see any significant differences in Arc or Egr-1 mRNA expression in garcinol treated rats compared to vehicle (*p* > 0.05; Figures 2E,F). These data suggest that systemic garcinol administration is capable of decreasing levels of IEG expression in the LA in rats receiving cocaine-cue memory reactivation, but it does not alter expression patterns in no reactivation controls.

### Systemic Garcinol Reduces Histone Acetylation in the LA of Reactivated Rats

**Figure 3:**
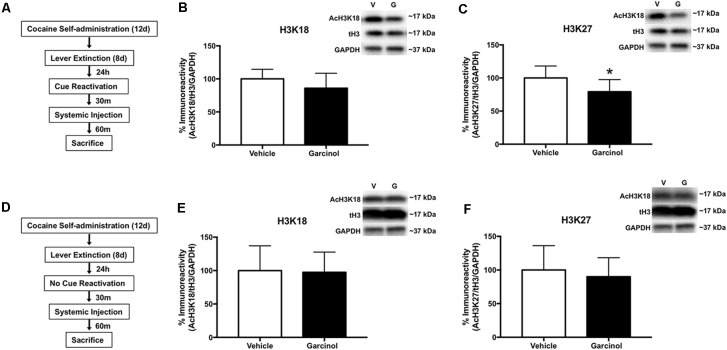
Systemic garcinol reduces histone acetylation in the LA of reactivated rats. **(A)** Schematic of the behavioral protocol. **(B)** Mean (±SEM) acetyl-H3K18 immunoreactivity from punches taken from the LA. Here acetyl-H3 protein levels have been normalized to total levels of H3 and GAPDH levels for each sample and expressed as a percentage of the vehicle group. **(C)** Mean (±SEM) acetyl-H3K23 immunoreactivity from punches taken from the LA analyzed as in **(B)**. *p < 0.05, significant decrease relative to vehicle group. **(D)** Schematic of the behavioral protocol. **(E)** Mean (±SEM) acetyl-H3K18 immunoreactivity from punches taken from the LA analyzed as in **(B)**. **(F)** Mean (±SEM) acetyl-H3K23 immunoreactivity from punches taken from the LA analyzed as in **(B)**. *p < 0.05.

In our next set of experiments, we sought to examine whether systemic garcinol (10 mg/kg, i.p.) administration resulted in molecular epigenetic changes in the LA. Garcinol is an inhibitor of the two HATs CBP/p300 and PCAF (Balasubramanyam et al., 2004). Further, previous studies have demonstrated that CBP/p300 actively is responsible for acetylating lysine residues 18 and 27 (K18 and K27) and that deletion of CBP/p300 in cells specifically reduces acetylation levels on these residues compared to others (Jin et al., 2011). We hypothesized that following reactivation of a cocaine-associated cue memory (but not in non-reactivated controls), levels of acetylated histone H3 K18 and K27 would be significantly decreased in the LA in response to systemic garcinol administration. For these experiments, rats received either vehicle or garcinol injection after memory reactivation (vehicle *N* = 7; garcinol *N* = 8; see Figure 3A) or no reactivation (vehicle *N* = 6; garcinol *N* = 7; see Figure 3D). There were no differences in total drug infusions between vehicle and garcinol treated rats in the reactivated and no reactivation group during the 12 days of cocaine self-administration training (*p* > 0.05; Supplementary Figure S2). Additionally, no differences were seen in the number of active lever presses during the 8 days of lever extinction between groups (*p* > 0.05; Supplementary Figure S2). Rats were then sacrificed 90 min after memory reactivation or no reactivation and Western blotting was performed on LA tissue. The results revealed a nonsignificant difference in AcH3 K18 (*p* > 0.05, Cohen’s *d* = 0.76; Figure 3B); however, levels of AcH3 K27 were significantly decreased in the garcinol group compared to the vehicle group in reactivated rats (*t*_(13)_ = 2.20, *p* < 0.05, Cohen’s *d* = 1.14; Figure 3C). In rats receiving no memory reactivation session, neither AcH3 K18 nor AcH3 K27 levels were altered in response to garcinol administration when compared to the vehicle group (*p* > 0.05; Figures 3E,F). These data suggest that systemic garcinol treatment following cocaine-cue memory reactivation is capable of decreasing levels of histone H3 acetylation in the LA.

### Systemic Inhibition of HDAC Activity Rescues the Reinstatement Impairment Induced by Garcinol

**Figure 4:**
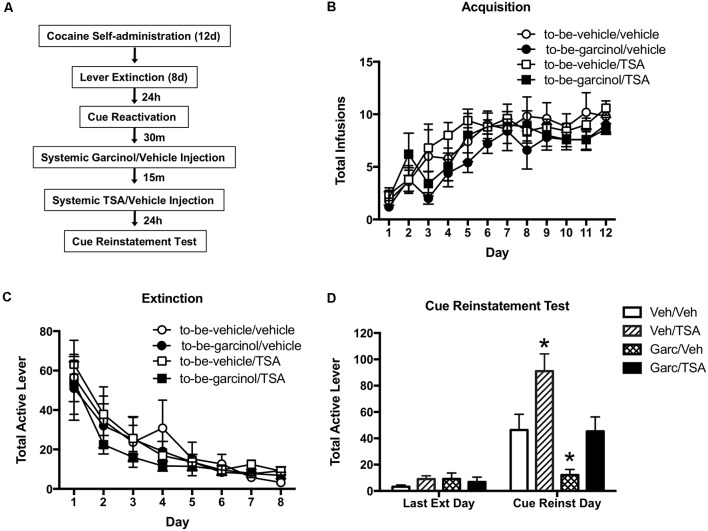
Systemic inhibition of histone deacetylase (HDAC) activity rescues the reinstatement impairment induced by garcinol. **(A)** Schematic of the behavioral protocol. **(B)** Total infusions per group across each day of cocaine self-administration. **(C)** Total active lever presses across each day of extinction. **(D)** Total active lever presses on the last day of extinction compared to during the cue-induced reinstatement test. *p < 0.05, Veh/trichostatin A (TSA) and Garc/Veh significant increase and decrease, respectively, relative to all other groups.

Previous studies utilizing a fear conditioning paradigm have reported that intra-LA infusion of the HDAC inhibitor TSA following training and following memory reactivation leads to an increase in histone H3 levels in the amygdala (Maddox and Schafe, 2011; Monsey et al., 2011). Further, it was shown that intra-LA TSA is capable of enhancing both the consolidation and reconsolidation of auditory fear memory (Maddox and Schafe, 2011; Monsey et al., 2011). In light of this, in our final set of experiments, we hypothesized that by using the HAT inhibitor garcinol as well as the HDAC inhibitor TSA we could bi-directionally regulate reinstatement (see Figure 4A).

There was no significant difference observed between to-be-vehicle and to-be-garcinol treated groups across the 12 days of cocaine self-administration (*p* > 0.05; Figure 4B). Likewise, there were no significant differences between groups across extinction (*p* > 0.05; Figure 4C). However, when comparing the last day of extinction and the cue reinstatement test day, a RM-ANOVA revealed a significant main effect of day (*F*_(1,16)_ = 224.30, *p* < 0.0001), group (*F*_(3,16)_ = 51.68, *p* < 0.0001) and day by group interaction (*F*_(3,16)_ = 36.68, *p* < 0.0001; Figure 4D). Bonferroni’s test comparing groups on the cue reinstatement day revealed a significant decrease in lever pressing in the Garcinol/TSA group compared to the Vehicle/TSA group (*p* < 0.0001). Further, there was a significant decrease in lever pressing in the Garcinol/Vehicle group when compared to the Garcinol/TSA group (*p* < 0.0001).

These data indicate that inhibiting HATs leads to an impairment in cocaine-cue memory reinstatement and conversely, inhibiting HDACs leads to an enhancement in the reinstatement of drug-seeking behavior. When given together TSA appears to rescue (or prevent) this garcinol-induced impairment in reconsolidation—confirming that altering levels of histone acetylation plays an important role in modulating reconsolidation processes.

## Discussion

In the present study, we sought to investigate the effects of the naturally-derived compound, garcinol, on downstream targets such as acetylation of histones and immediate-early gene expression in the amygdala. Here, we also examined whether using modulators of histone acetylation would alter the reinstatement of a cocaine-cue memory. We identified the LA as an important structure responding to garcinol, as intra-LA infusion of this compound following cocaine-cue memory reactivation was sufficient to block the reconsolidation of this memory. This impairment was isolated to only those memories that were reactivated as we did not observe any memory deficits in our non-reactivated controls. Following the systemic injection of garcinol after memory reactivation, we observed a decrease in expression of the IEGs Arc and Egr-1 in the LA as well as a decrease in histone H3 K27 acetylation. Finally, we showed that systemic injection of the HDAC inhibitor TSA is capable of increasing reinstatement of a cocaine-associated memory; however, when given in conjunction with garcinol, this effect is prevented and reinstatement is reduced to baseline levels. Collectively, these data are consistent with our previous findings that characterize garcinol as a potentially clinically useful amnestic agent to treat mnemonic pathologies such as substance use disorder (Monsey et al., 2017).

Prior studies established garcinol’s ability to impair a cocaine-associated memory in a manner that is specific to reactivated memories only, long-lasting, cue-specific, temporally-constrained, and persist following extended cocaine access when administered systemically after cue reactivation (Monsey et al., 2017). Additionally, it has been shown that garcinol can impair reconsolidation when given systemically following a US (cocaine) reactivation session and that garcinol’s effects are only observed if administered during the labile period following memory reactivation (Dunbar and Taylor, 2017a). While these studies hold promise for clinical utility due to the systemic administration of garcinol, it is also of importance to identify brain region-specific sites where garcinol could be exerting its effects on mnemonic processing. We chose to examine the LA due to its involvement in the formation and storage of emotionally salient associative memories (Sorg, 2012; Torregrossa and Taylor, 2013, 2016; Taylor and Torregrossa, 2015). Our observation that intra-LA garcinol infusion is sufficient to impair the reinstatement of a cocaine-cue memory suggests that this may be one target brain region being affected following systemic administration. In agreement with previous data, this effect was constrained only to memories that had been reactivation because we did not observe a reconsolidation impairment in our non-reactivated controls following intra-LA garcinol infusion. Garcinol’s precise mechanisms of action that might contribute to its ability to impair the reconsolidation of a cocaine-cue memory following reactivation remain uncertain. In light of this and our previous data, we also examined potential downstream molecular modifications known to be altered in response to garcinol.

The results of our molecular experiments confirmed that systemic garcinol does indeed alter levels of mRNA expression and histone acetylation in the LA when administered after memory reactivation. We first examined the expression of the immediate-early genes Arc and Egr-1 and found that mRNA levels of both IEGs were reduced in garcinol injected rats. This cascade of IEGs is important as induction of IEG expression in brain regions occurs during cognitive processing such as neuronal activation during behavioral tasks (Guzowski et al., 2001; Ziółkowska et al., 2011; Minatohara et al., 2015; Li et al., 2016). Both Arc and Egr-1 have been previously reported to play a critical role in consolidation and reconsolidation processes of associative memories (Ploski et al., 2008; Maddox et al., 2010; Maddox and Schafe, 2011; Ziółkowska et al., 2011; Alaghband et al., 2014). One study utilizing a mouse model of drug self-administration found that cue-induced reinstatement of cocaine-seeking lead to an induction of Arc and Egr-1 expression in the medial prefrontal cortex as well as the amygdala (Ziółkowska et al., 2011). Others have reported that intra-LA infusion of Egr-1 antisense oligodeoxynucleotides prior to a cue-induced reinstatement test abolishes reinstatement and cocaine-seeking behavior (Lee et al., 2006). Further, Arc knockout mice exhibit impairments in long-term memory despite intact short-term memory formation and also show negative alterations in long-term potentiation and long-term depression (Plath et al., 2006). Thus, garcinol’s ability to reduce the expression of these IEGs after memory reactivation may be one way in which it exerts its effects and impairs cocaine-cue memory reconsolidation.

Previous research has established that epigenetic modulation of histone acetylation in the LA also contributes to the reconsolidation process in other models of memory formation such as fear conditioning (Maddox and Schafe, 2011; Maddox et al., 2013a,b; Monsey et al., 2015). One study using a fear memory reconsolidation paradigm revealed that levels of histone H3 acetylation were significantly elevated following memory reactivation and that HAT inhibitors such as garcinol and c646 were capable of diminishing these reactivation-related increases and impaired reconsolidation (Maddox and Schafe, 2011; Maddox et al., 2013a,b). Others have shown similar mechanisms are crucial for responses following cocaine self-administration. It has been reported that changes in histone H3 acetylation in the striatum and nucleus accumbens are essential for cocaine-induced neuroplasticity in addition to motivation for the reinforcing effects of cocaine (Kumar et al., 2005; Wang et al., 2010). In agreement with this, we also observed the regulation of histone acetylation in response to memory reactivation in our own experiments. We report a decrease in histone H3 K27 acetylation in the LA following memory reactivation and systemic garcinol administration. Epigenetic alterations and posttranslational modifications such as histone acetylation have been widely implicated in the pathogenesis of numerous psychiatric diagnoses including depression, PTSD, schizophrenia, Rett syndrome, and addiction (Tsankova et al., 2004, 2006, 2007; Kumar et al., 2005; Maddox and Schafe, 2011; Monsey et al., 2011, 2017; Nott et al., 2016; Thomas, 2017). Acetylation of histones occurs on lysine residues on the protein tail. Increasing levels of histone acetylation shift the chromatin structure toward an open state, allowing access for transcriptional machinery and thus enhances gene transcription. Conversely, a reduction in histone acetylation is thought to lead to a compact form of chromatin and subsequent transcriptional repression. In the present study, we observed a decrease in acetylation of histone H3 K27, but not of H3 K18, following memory reactivation and garcinol treatment, which is consistent with garcinol’s role as a HAT inhibitor. We initially predicted that garcinol would decrease acetylation of both of these lysine residues, however, we may have failed to observe a difference in H3 K18 expression due to low power in this experiment. Further studies including more animals and the examination of acetylation changes on other lysine residues might yield alternate results. We hypothesize that acetylation is reduced on other lysine residues in response to garcinol as well. This, in turn, could lead to a decrease in mRNA transcription as we observed with Arc and Egr-1 in the present set of experiments. It would be of interest to explore this theory further and examine acetylation levels on promoter regions of these IEGs as well as on other late-phase genes known to underlie the reconsolidation process. More comprehensive measures of examining changes in acetylation on proteins regulated by cocaine-cue memory reactivation and garcinol administration using proteomic analysis would also contribute to our understanding of the role these epigenetic modifications play in our behavioral paradigm (see Rich et al., 2016; Torregrossa et al., 2019).

In our final set of experiments, we built on the hypothesis that changing levels of histone acetylation would result in altered levels of reinstatement of a cocaine-associated memory and drug-seeking behavior. For these experiments, we investigated the effects of garcinol as well as the HDAC inhibitor TSA both alone and given in conjunction. Previous studies have shown that intra-LA infusion of TSA elevates levels of histone H3 K9/14 in the amygdala and enhances the consolidation and reconsolidation of auditory fear memory (Maddox and Schafe, 2011; Monsey et al., 2011). Interestingly, when TSA was given alone following a cue reactivation session we also observed an enhancement in reinstatement, consistent with the notion that levels of histone acetylation may contribute to changes in the reconsolidation process. Conversely, when garcinol and TSA are administered together following memory reactivation reinstatement returns to baseline levels; therefore, it appears that TSA can prevent the garcinol-induced impairment in reconsolidation observed in our initial studies. This suggests that inhibiting both HATs and HDACs together might result in subthreshold changes in histone acetylation levels and thus have no outcome on behavior in our specific paradigm. Further testing is required to more thoroughly dissect the interplay between garcinol and TSA and how they both individually and collectively contribute to modifications to cocaine-associated memory reconsolidation and drug-seeking behavior.

In summary, the results of the present study provide further support for garcinol as a promising pharmacological tool for impairing reconsolidation of an appetitive cocaine-associated cue memory and that one avenue of action may be through changes in histone acetylation in the LA. Thus, epigenetic modulation of memory-related gene expression and the role histone acetylation plays in memory reconsolidation—particularly in the LA—warrant further study. Examining these mechanisms may reveal the processes involved in the maintenance of drug-associated memories that disrupt sustained abstinence.

## Data Availability Statement

The datasets generated for this study are available on request to the corresponding author.

## Ethics Statement

The animal study was reviewed and approved by Yale University IACUC.

## Author Contributions

MM designed study, conducted experiments, data analysis, manuscript writing, and editing. SR assisted with conducting experiments, data analysis, and manuscript editing. JT assisted with study design, interpretation of results, and manuscript editing.

## Conflict of Interest

The authors declare that the research was conducted in the absence of any commercial or financial relationships that could be construed as a potential conflict of interest.

## Supplementary Material

FIGURE S1 Behavioral data for examination of immediate-early gene expression in the LA (Figure 2). **(A)** Schematic of the behavioral protocol for reactivated rats. **(B)** Total infusions per group across each day of cocaine self-administration. **(C)** Total active lever presses across each day of extinction. **(D)** Schematic of the behavioral protocol for non-reactivated rats. **(E)** Total infusions per group across each day of cocaine self-administration. **(F)** Total active lever presses across each day of extinction.

FIGURE S2 Behavioral data for examination of histone acetylation in the LA (Figure 3). **(A)** Schematic of the behavioral protocol for reactivated rats. **(B)** Total infusions per group across each day of cocaine self-administration. **(C)** Total active lever presses across each day of extinction. **(D)** Schematic of the behavioral protocol for non-reactivated rats. **(E)** Total infusions per group across each day of cocaine self-administration. **(F)** Total active lever presses across each day of extinction.
