# Supplementary figures and images for: Regulation of Garcinol on Histone Acetylation in the Amygdala and on the Reconsolidation of a Cocaine-Associated Memory
Source: Front Behav Neurosci. 2020 Jan 8;13:281. doi: 10.3389/fnbeh.2019.00281 (PMC6961612; doi:10.3389/fnbeh.2019.00281)

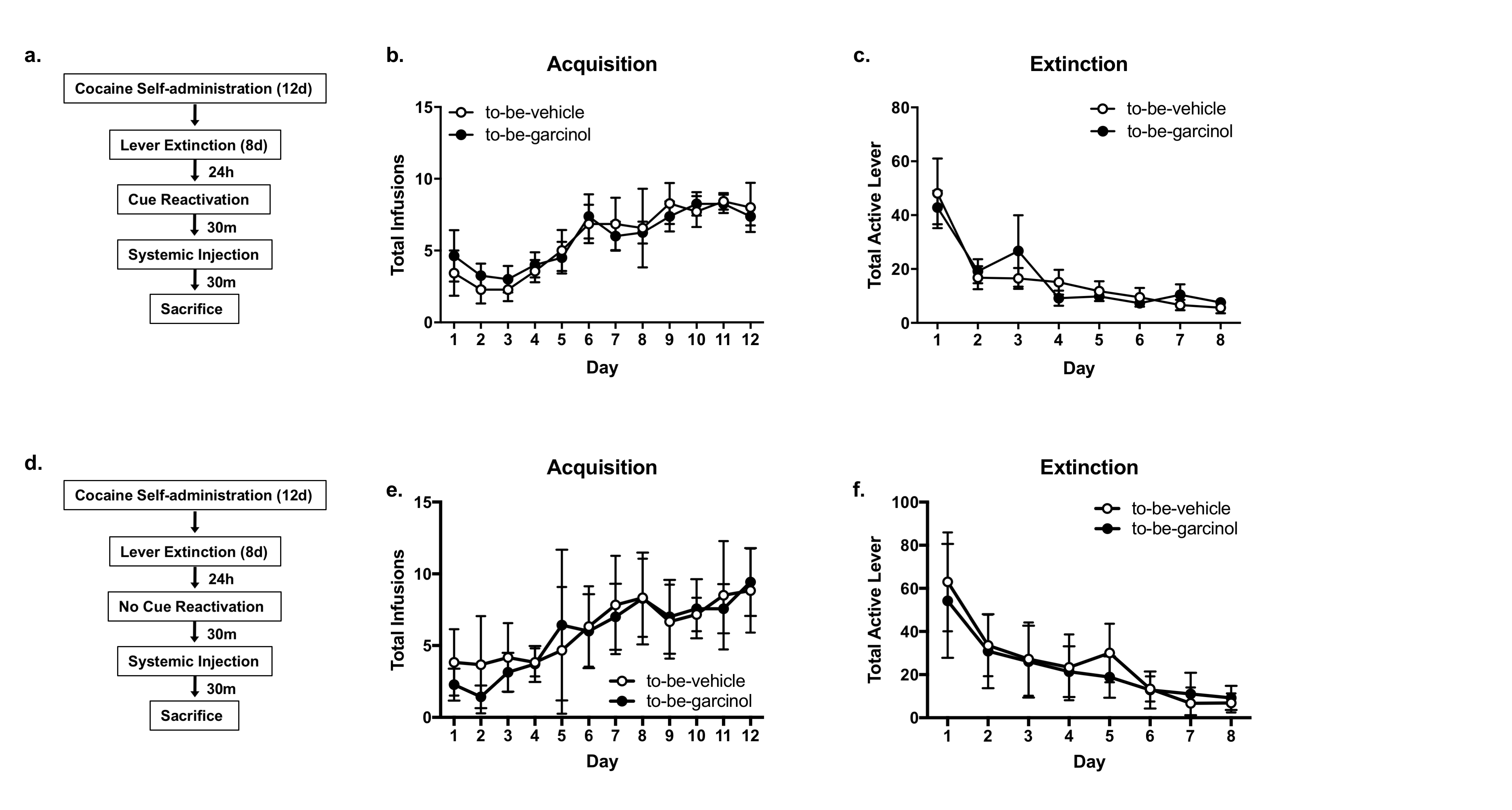

Supplement: FIGURE S1 — Behavioral data for examination of immediate-early gene expression in the LA (Figure 2). (A) Schematic of the behavioral protocol for reactivated rats. (B) Total infusions per group across each day of cocaine self-administration. (C) Total active lever presses across each day of extinction. (D) Schematic of the behavioral protocol for non-reactivated rats. (E) Total infusions per group across each day of cocaine self-administration. (F) Total active lever presses across each day of extinction. [file Image_1.TIFF]

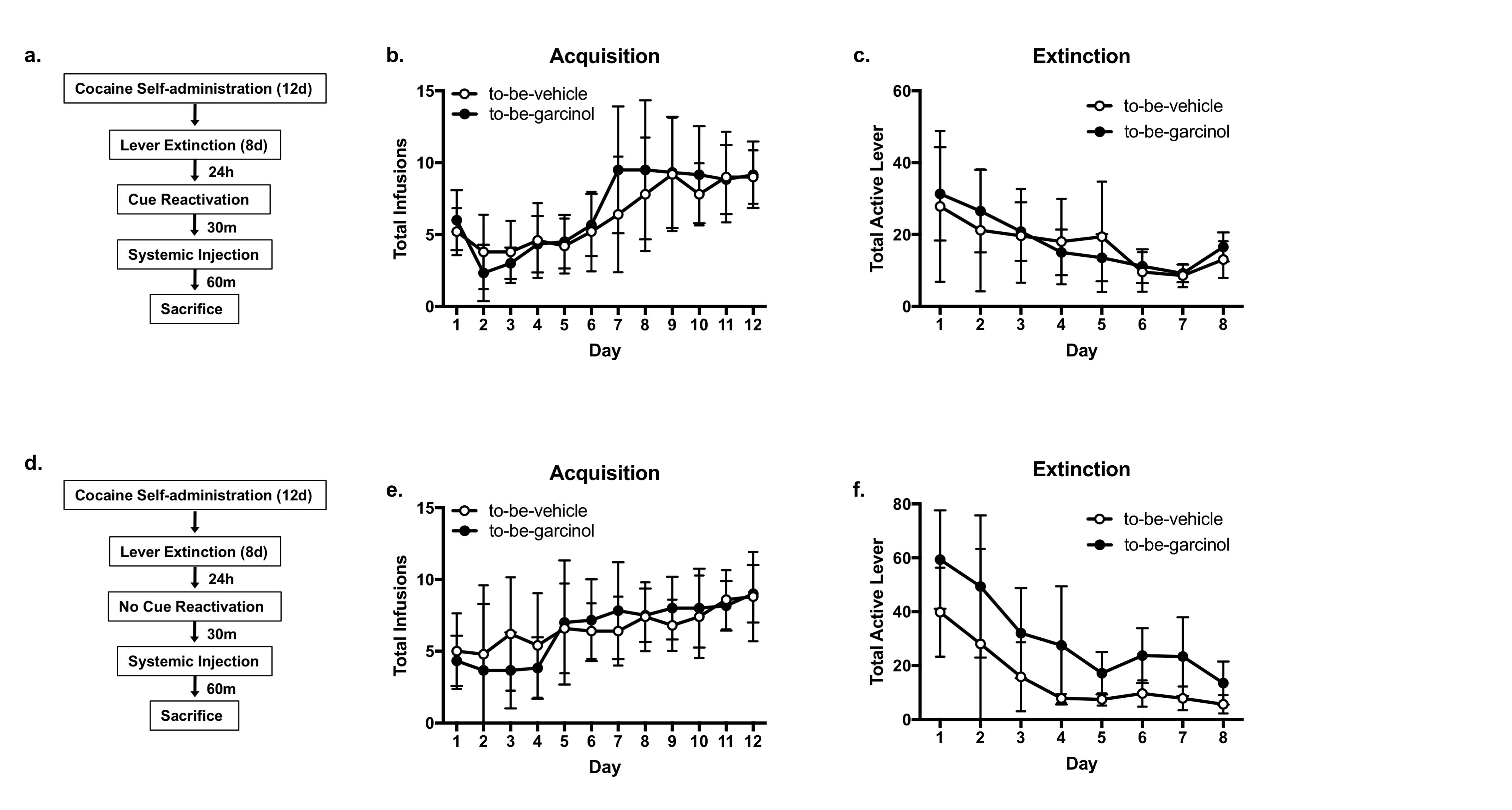

Supplement: FIGURE S2 — Behavioral data for examination of histone acetylation in the LA (Figure 3). (A) Schematic of the behavioral protocol for reactivated rats. (B) Total infusions per group across each day of cocaine self-administration. (C) Total active lever presses across each day of extinction. (D) Schematic of the behavioral protocol for non-reactivated rats. (E) Total infusions per group across each day of cocaine self-administration. (F) Total active lever presses across each day of extinction. [file Image_2.TIFF]
